# Tardive Dyskinesia in a Postoperative Gynecological Patient After Single Dose Administration of Metoclopramide

**DOI:** 10.7759/cureus.18260

**Published:** 2021-09-24

**Authors:** Christina S Wong, Kyler W Perry, Amy Rosenbaum

**Affiliations:** 1 College of Osteopathic Medicine, Touro University Nevada, Henderson, USA; 2 Obstetrics and Gynecology, Women’s Health Associates of Southern Nevada, Las Vegas, USA

**Keywords:** gynecologic surgery, dopamine receptor blocking agent, metoclopramide, extrapyramidal symptom, tardive dyskinesia

## Abstract

Metoclopramide is a dopamine D2-receptor blocking agent commonly used to treat nausea, vomiting, and gastroparesis. Due to their mechanism of action, these drugs can lead to extrapyramidal side effects such as tardive dyskinesia. In this article, we report a case of a nulliparous gynecology patient who developed dyskinetic movements after intraoperative administration of metoclopramide. During further workup after stabilization, she was found to have several risk factors for tardive dyskinesia. As the occurrence of this phenomenon is somewhat rare, this case report aims to discuss the condition, associated risk factors, and differentiation from other diagnoses.

## Introduction

Tardive dyskinesia (TD) is a potentially permanent and persistent syndrome of abnormal involuntary movements with delayed onset secondary to blockage of dopamine D2 receptors [1]. These movements involve the face, mouth, tongue, trunk, and extremities and are often rhythmic, repetitive, and stereotypic in nature [2]. TD most commonly occurs in individuals being treated with dopamine-receptor blocking agents (DRBAs) [1,3]. Metoclopramide, a DRBA, is often used to treat a variety of gastrointestinal symptoms, including chronically for gastroparesis and acutely for nausea and vomiting. Previous studies have shown the development of TD in patients on chronic metoclopramide for gastroparesis [3]. However, there have only been a few case reports of TD in patients using metoclopramide acutely for nausea and vomiting [4-6].

## Case presentation

A 26-year-old nulliparous Asian female with a medical history significant for anxiety and depression presented for an elective diagnostic laparoscopy for pelvic and perineal pain due to suspected endometriosis. She was not taking any medications, and the documented allergies included gluten, dairy, and amoxicillin. A pre-operative ultrasound one week prior displayed a retroverted uterus with a smooth contour and normal echotexture. Endometrium measured 14 mm, and ovaries were normal. There was a minimal amount of free fluid noted, but no other irregularities were observed. All preoperative labs and EKG were normal. Risks and benefits of surgery were discussed, as well as alternative treatments. The patient was agreeable to surgery, and all questions were answered.

On the day of surgery, during evaluation by anesthesia, the patient noted that she had experienced daytime sleepiness and possible CNS pathology in the past. Upon further questioning, she revealed a history of syncopal events over the course of the previous year. She was referred to cardiology and diagnosed with orthostatic hypotension. She was advised to hydrate after vigorous workouts and had not experienced any pre-syncopal or syncopal events since then. With regard to the vague history of possible CNS pathology, the patient denied any proven overt seizure activity. She did not see a neurologist because her symptoms resolved and had been cleared for the diagnostic laparoscopy by her primary physician.

Upon arrival at the operating room at 15:21, standard monitoring devices were applied. The patient was subsequently sedated and intubated at 15:33. During this time, she was given 4 mg ondansetron, 4 mg dexamethasone, 10 mg metoclopramide, 20 mg famotidine, 5 mL lidocaine, 50 mcg fentanyl, 200 mg propofol, and 80 mg succinylcholine intravenously (IV). At 15:44, the anesthesiologist performed peripheral nerve transversus abdominis plane (TAP) blocks using 20 mL of ropivacaine 0.5% injections and then administered rocuronium 30 mg IV. The surgery began at 15:53. The overall operative course was uneventful, and the patient received an additional 50 mg propofol and 2 mg hydromorphone IV during the surgery. The patient emerged from anesthesia at 16:26, at which point 2 mg midazolam, 0.5 mg glycopyrrolate, and 3 mg neostigmine were administered IV. A detailed list of medications administered during the surgery can be seen in Table 1. The patient arrived at the post-anesthesia care unit (PACU) at 16:40.

**Table 1:** Medication and time of administration during the operative course. IV: intravenously, PF: preservative free, MS: methylsulfate

Medication	15:33	15:44	16:12	16:26	Total
Ondansetron 4 mg/2 mL vial IV	4 mg				4 mg
Dexamethasone 4 mg/mL 1 mL vial IV	4 mg				4 mg
Metoclopramide 5 mg/mL injection 2 mL IV	10 mg				10 mg
Famotidine 20 mg/2 mL injection IV	20 mg				20 mg
Lidocaine 2% PF injection 10 mL IV	5 mL				5 mL
Fentanyl 250 mcg/5 mL injection IV	50 mcg				50 mcg
Propofol 200 mg/20 mL IV	150 mg		50 mg		200 mcg
Succinylcholine 20 mg/mL vial 10 mL IV	80 mg				80 mg
Rocuronium 10mg/mL vial 5 mL IV		30 mg			30 mg
Ropivacaine 0.5% vial 30 mL, local infiltrate		20 mL			20 mL
Hydromorphone 2 mg/mL injection IV			2 mg		2 mg
Midazolam 1 mg/mL injection 5 mL IV				2 mg	2 mg
Glycopyrrolate 0.2 mg/mL 5 mL vial IV				0.6 mg	0.6 mg
Neostigmine MS 1 mg/mL vial 10 mL IV				3 mg	3 mg

Postoperative hospital course

She received 10 mg Reglan (metoclopramide) during sedation and intubation. She subsequently developed truncal dyskinetic movements and agitation immediately after emergence from anesthesia in the operating room. No medication was administered for her presenting symptoms at this time. The patient was transferred to the post-anesthesia care unit (PACU), where abnormal movements became more pronounced with significant truncal and upper and lower extremity movements with waxing and waning mentation. The patient was able to open her eyes to verbal commands, and the event lasted between 30 to 60 minutes despite the administration of 25 mg Benadryl (diphenhydramine) at 17:05 and 2 mg Ativan (lorazepam) at 17:12. Due to the lack of response to these medications and persistent dyskinetic activity, the rapid response team was called due to concerns for airway protection. She was subsequently intubated, sedated, and admitted to the intensive care unit (ICU). The right femoral central vein was cannulated for venous access, and 20 mg etomidate and 20 mg rocuronium were given prior to this procedure. The patient was also given 1 g Keppra (levetiracetam) for seizure prophylaxis, as the underlying cause for her presentation was still unknown. Workup with a computerized tomography scan (CT) of her head was unremarkable and showed no signs of possible infarction. An electroencephalogram (EEG) showed mild bihemispheric slowing, but no epileptiform discharges were noted.

The patient was extubated after 24 hours and had returned to her baseline by that time. She denied any recollection of the event other than hearing the call for rapid response. Additional medical history was obtained and included suspected low blood pressure, which had been evaluated by cardiology, and a questionable seizure disorder. She reported a recent history (five months prior) of confusion, lower extremity shaking, and loss of consciousness that was witnessed by her mother. She did not seek medical attention then. She had two additional episodes since that time, with loss of consciousness, confusion, and dizziness but without any serious injuries. She also reported a remote history of a concussion two years prior.

Laboratory measurements prior to discharge were significant for leukocytosis of 12,200/mcL with elevated neutrophils (9,660/mcL). The patient also had an elevated total bilirubin of 3.0 mg/dL and direct bilirubin of 1.0 mg/dL.

Discharge and outpatient follow-up

The patient was discharged after 48 hours. She was given midodrine 5 mg for her low blood pressure and was told to continue levetiracetam 500 mg three times a day. She was also advised against driving until cleared by neurology. The patient remained stable during her postoperative obstetrics and gynecology (OBGYN) appointment and underwent an outpatient neurology workup.

## Discussion

Tardive dyskinesia (TD) is a disorder of abnormal muscle movements that is more commonly observed in older individuals with chronic use of DRBAs [1-3]. Interestingly, there have been a few cases of acute TD and idiosyncratic drug reactions in young patients using DRBAs [4-6]. In one such case report, a 16-year-old male developed TD after two days of oral metoclopramide use [5]. Here we present a case of a 26-year-old Asian female who developed TD acutely after a single 10 mg dose of IV metoclopramide at the time of sedation and intubation. Risk factors pertaining to our patient for the development of TD include female gender and known history of CNS pathology [7,8].

Metoclopramide antagonizes dopamine D2 receptors in the basal ganglia, which is the mechanism of action leading to side effects like tardive dyskinesia, as well as other extrapyramidal symptoms such as acute dystonia [6]. First-line treatments of TD include VMAT2 inhibitors such as deutetrabenazine and valbenazine, discontinuation of the offending agent, and use of diphenhydramine in acute settings [9]. Our patient received diphenhydramine and lorazepam shortly after the onset of dyskinetic movements; however, her symptoms persisted, requiring intubation for airway maintenance and transfer to intensive care. Further workup with an EEG and CT head suggested no underlying seizure activity or other CNS pathology that could have prompted the dyskinetic episode.

While this case does present like tardive dyskinesia, it is important to consider other possible diagnoses. In acute settings, it is often difficult to differentiate between TD and an acute dystonic reaction, both of which can be caused by metoclopramide. Generally speaking, dystonia often presents with acute use of DRBAs [4]. TD symptoms are more often seen with chronic use of DRBAs, but rare incidences of its development with acute use have been noted in the literature as well [1]. While this complicates the formulation of a diagnosis, our patient had many characteristics of TD, pushing us in that direction. Dystonia typically presents as prolonged muscle contractions in specific muscles, leading to abnormal posturing and spasms. These contortions are often painful, and onset occurs when levels of the offending drug begin to decline. Conversely, TD presents as involuntary painless muscle movements in large muscle groups, such as the face, limbs, and trunk. These movements are smooth, repetitive, and not prolonged [10]. Tardive dyskinesia and acute dystonic reactions are also treated similarly, further challenging patient workup [6]. Standard treatment for acute dystonia is benztropine and diphenhydramine, while TD treatment includes VMAT2 inhibitors and the use of diphenhydramine in acute settings [9].

There are multiple possible diagnoses that a patient with the development of acute extrapyramidal symptoms could have, including TD, acute dystonia, or simply an idiosyncratic drug reaction [4]. Ultimately, our patient was discharged with the diagnosis of tardive dyskinesia. She met several known risk factors, including her gender and history of prior CNS-associated events. Limitations to this case report can be seen with the inability to confirm the diagnosis. Active seizures were ruled out with a negative CT head and EEG that only showed mild bihemispheric slowing, but it is difficult to conclusively rule out an acute dystonic reaction. Further, with the persistence of her dyskinetic movements after administration of diphenhydramine, she was subsequently intubated due to concerns for airway patency. Other underlying neurologic diseases also cannot be ruled out, even with a negative workup. This, however, presents the question of how to correctly diagnose future cases that present similarly. Nevertheless, we present a unique case of diagnosed tardive dyskinesia after a single dose of metoclopramide in the intraoperative setting.

## Conclusions

Tardive dyskinesia is an exceedingly rare complication of acute metoclopramide use, especially in younger patients receiving a single therapeutic dose. In this article, we report a case of a nulliparous gynecology patient who was diagnosed with tardive dyskinesia after intraoperative administration of metoclopramide to avoid postprocedural nausea and vomiting. Workup was significant for several risk factors associated with the development of this extrapyramidal symptom. It is important for healthcare providers to quickly identify patient-specific risk factors for the development of tardive dyskinesia and other extrapyramidal symptoms and approach the use of metoclopramide and other dopamine D2-receptor blocking agents with caution in the presence of these circumstances. Should associated symptoms arise with the use of these medications, heightened awareness will allow for appropriate treatment and patient care.
